# BPA as a Mammary Carcinogen: Early Findings Reported in Rats

**DOI:** 10.1289/ehp.121-a284

**Published:** 2013-09-01

**Authors:** Lindsey Konkel

**Affiliations:** Lindsey Konkel is a Worcester, MA–based journalist who reports on science, health, and the environment. She writes frequently for *Environmental Health News* and *The Daily Climate*.

Perinatal exposure of rodents to low doses of bisphenol A (BPA) has been associated with altered mammary gland development and increased propensity for mammary tumors later in life.^1^ A study by Tufts University researchers in the current issue of *EHP* suggests that BPA may act as a malignant mammary gland carcinogen in rats at internal doses comparable to those seen in humans.^2^

Prenatal exposures to chemicals can yield different results than studies in adult animals.^3^ Previous studies have shown that rats and mice exposed prenatally to both BPA and a known carcinogen in adulthood grew more tumors faster than animals exposed only to the carcinogen.^4^ In a 2007 study, senior author Ana Soto and colleagues detected intraductal hyperplasias—overgrowths of cells considered to be precursors to mammary gland carcinomas in both rodents and humans—at postnatal day 50 and 95 in Wistar Furth rats exposed prenatally to environmentally relevant levels of BPA.^5^ Some of these animals formed ductal carcinoma *in situ* (DCIS)—localized tumors that sometimes but not always progress to malignancy.

According to the authors, the current study is the first to report malignant tumor formation following developmental exposure to environmentally relevant levels of BPA. Soto, a cancer researcher at the Tufts University School of Medicine, calls the findings unexpected. “We were measuring response to internal dose and looking for preneoplastic lesions. Instead, what we saw were full-blown tumors,” she says.

Soto and colleagues treated pregnant Sprague-Dawley rats with varying amounts of BPA during gestation and lactation or during gestation only. They measured levels of total BPA and unconjugated or free BPA in the blood of pregnant dams and their fetuses and nursing pups. Unconjugated BPA in blood is considered the hormonally active form of the chemical.

They detected total and unconjugated BPA in all the treated dams and their fetuses and in one-third of the nursing pups exposed to 250 µg BPA per kg body weight per day via subcutaneous pump. Exposure of dams to a BPA dose of 250 µg/kg BW/day either during gestation or during gestation and lactation resulted in an average blood level of unconjugated BPA of 1.25 ng/mL. (By comparison, a 2010 review of human biomonitoring studies reported mean blood levels of BPA in healthy adults of approximately 1 ng/mL.^6^)

The researchers observed six mammary gland tumors in female offspring exposed perinatally to BPA at doses ranging from 0.25 to 250 µg/kg BW/day as early as 90 days after birth. Five of the tumors were classified as adenocarcinomas—malignant tumors with the potential to be invasive and metastatic.

No malignant tumors were detected in any of the control animals. However, because there were so many more exposed rats than controls (230 versus 65), the investigators cannot rule out the possibility that the observed tumors developed spontaneously, despite prior evidence that female Sprague-Dawley rats rarely form spontaneous malignant tumors before 210 days of age.^7^^,^^8^

**Figure 1:**
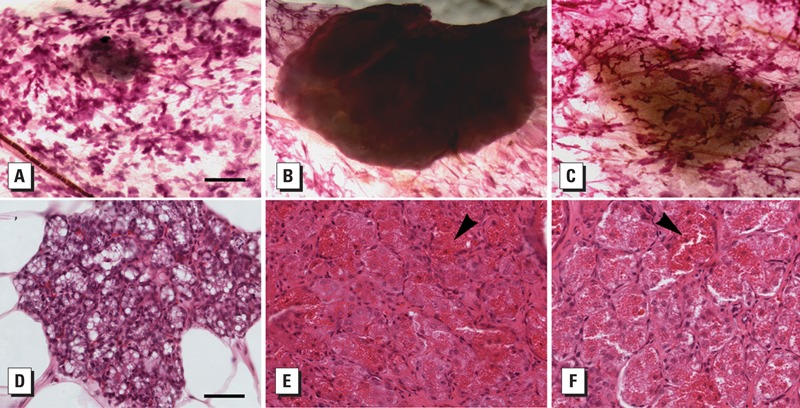
Micrographs show lesions detected in mammary gland tissue from female rats exposed to varying doses of BPA either during gestation only or during gestation and lactation. The findings suggest BPA may be a malignant mammary carcinogen, but the study must be replicated using more animals before any firm conclusions can be drawn. Acevedo et al./http://dx.doi.org/10.1289/ehp.1306734

Epidemiological studies have suggested that exposure to elevated estrogen levels in the womb may be associated with an increase in a woman’s lifetime risk of breast cancer.^9^^,^^10^ Exposure to the estrogen mimic BPA is widespread, with one report from the nationally representative National Health and Nutrition Examination Survey (NHANES) finding that 90% of respondents over age 6 years had detectable levels of BPA in their urine.^11^ (NHANES, like most biomonitoring studies, measures only urinary levels of total and free BPA, not blood levels.^12^)

“From the point of view of human health research, the most notable aspect of the study is that the blood levels are very comparable to those found in humans, which isn’t always the case with animal studies,” says epidemiologist Barbara Cohn, director of the Oakland, California–based Child Health and Development Studies, a cohort of more than 15,000 mothers, daughters, and granddaughters aimed at deciphering the role that environmental exposures play in the development of diseases such as breast cancer.^13^

“This finding may be of great public health significance,” Cohn says. “But without a question it must be replicated before we can know for sure whether BPA is a complete carcinogen.” She says information on the impact of developmental exposure to BPA on breast cancer in humans will also be critical for understanding the public health significance of the findings.
